# The Cellular and Viral circRNAs Induced by Fowl Adenovirus Serotype 4 Infection

**DOI:** 10.3389/fmicb.2022.925953

**Published:** 2022-06-02

**Authors:** Xiao-Na Liu, Xiao-Ran Guo, Ying Han, Tian Tian, Jian Sun, Bai-Shi Lei, Wu-Chao Zhang, Wan-Zhe Yuan, Kuan Zhao

**Affiliations:** ^1^College of Veterinary Medicine, Hebei Agricultural University, Baoding, China; ^2^Hebei Veterinary Biotechnology Innovation Center, Hebei Agricultural University, Baoding, China

**Keywords:** FAdV-4, cellular circRNAs, viral circRNAs, miRNA, virus replication

## Abstract

Circular RNAs (circRNAs) are a new class of noncoding RNAs that play vital roles in many biological processes. Virus infection induces modifications in cellular circRNA transcriptomes and expresses viral circRNAs. The outbreaks of Hydropericardium-hepatitis syndrome (HHS) caused by fowl adenovirus serotype 4 (FAdV-4) have resulted in huge economic losses to the poultry industry worldwide. To investigate the expression of circRNAs during FAdV-4 infection, we performed transcriptome analysis of FAdV-4-infected leghorn male hepatoma (LMH) cells. In total, 19,154 cellular circRNAs and 135 differentially expressed (DE) cellular circRNAs were identified. The characteristics of the DE cellular circRNAs were analyzed and most of them were related to multiple biological processes according to GO and KEGG enrichment analysis. The accuracy of 10 cellular circRNAs were verified by semiquantitative RT-PCR and sequencing. The change trend was consistent with the RNA sequencing results. Moreover, 2014 viral circRNAs were identified and 10 circRNAs were verified by the same methods. Our analysis showed that seven circRNAs with the same 3′ terminal and variable 5′ terminal regions were located at pTP protein and DNA pol protein of FAdV-4, which may be generated *via* alternative splicing events. Moreover, the expression level of viral circRNAs was closely related to the replication efficiency of the virus and partial of the viral circRNAs promoted the replication of FAdV-4. Competing endogenous RNA analysis further showed that the effects of cellular and viral circRNAs on host or viral genes may act *via* miRNAs. Collectively, our findings first indicate that FAdV-4 infection induced the differential expression of cellular circRNAs and FAdV-4 also expressed viral circRNAs, some of which affected FAdV-4 replication. These findings will provide new clues for further understanding FAdV-4 and provide a basis for investigating host-virus interactions.

## Introduction

Hydropericardium-hepatitis syndrome (HHS) is an important emerging disease, which is responsible for huge economic losses to poultry industry all over the world. It mainly caused by fowl adenovirus serotype 4 (FAdV-4), a hepatotropic virus that causes severe hepatic damage. HHS was first reported in the Ankara region near Karachi, Pakistan in 1987 and also named as the ‘Ankara disease’ (Anjum et al., 1989). FAdVs are non-enveloped viruses with double-stranded DNA, belonging to the family *Adenoviridae, aviadenovirus* (Hess, 2013). Based on restriction enzyme digestion patterns and serum cross-neutralization tests, FAdVs are divided into five species (FAdV-A to FAdV-E) with 12 serotypes (FAdV-1 to 8a and 8b to 11; Hess, 2000). FAdV-4, which causing the sudden onset of disease with high mortality in infected chickens, is often transmitted horizontally *via* the fecal-oral route. The main pathological changes include hepatomegaly, accumulation of transparent or light-yellow exudates in the pericardium, friability and yellow discoloration of the liver (Balamurugan and Kataria, 2006; Sun et al., 2019). It has been found and reported in many countries (Liu et al., 2020; Xie et al., 2021) and has resulted in enormous economic loss to the poultry industry worldwide.

Eukaryotic cells can generate several classes of noncoding RNAs (ncRNAs), such as small ncRNAs (sncRNAs) and lncRNAs (Zhang et al., 2019). As a key member of non-coding RNA, circRNAs are a newly identified class of ncRNAs that have recently elicited increasingly attention (Memczak et al., 2013; Wilusz and Sharp, 2013). CircRNAs are formed by the back-splicing of a downstream splice donor site to an upstream splice acceptor site, thus producing a covalently closed RNA molecule (Li et al., 2018). They are remarkably stable, presumably because they lack free ends and are resistant to exonuclease activity (Lasda and Parker, 2014). Recent studies have shown that circRNAs can act as microRNA (miRNA) sponges. Numerous circRNAs possess lots of miRNA-binding sites, so they can sponge miRNAs efficiently to upregulate the expression of the genes inhibited by miRNAs (Hansen et al., 2013). Furthermore, emerging lines of studies have revealed that some circRNAs played important roles under physiological and pathological conditions, and they may serve as diagnostic or predictive biomarkers of diseases and provide new potential therapeutic targets (Tang et al., 2021).

Viral infections can induce modifications in cell transcriptomes, including the circRNA transcriptome. The expression pattern of host circRNAs induced by viruses had been investigated previously and the host circular RNAs were also found to be involved in antiviral signaling pathway (Tagawa et al., 2018; Li et al., 2021). Besides, during the infection, virus can also encode circRNA by itself. Several research teams have discovered and validated the presence of viral circRNAs in DNA viruses such as Epstein–Barr virus (EBV), Kaposi’s sarcomaassociated herpesvirus (KSHV) and human papillomaviruses (HPVs; Toptan et al., 2018; Ungerleider et al., 2018; Zhao et al., 2019). In addition, Cai et al. had predicted some circRNAs expressed by single-stranded RNA viruses by systematic bioinformatics analysis of the viral infection-related RNA sequencing (RNA-seq; Cai et al., 2021). What is more, some RNA viruses were validated to express the circRNAs, including respiratory syncytial virus (RSV) and Reovirus of grass carp reovirus (GCRV; Pan et al., 2021; Yao et al., 2021). The viral circRNA possesses multiple biological roles including the regulation of viral replication or persistence and host immune evasion (Tycowski et al., 2015).

However, the identification and characterization of viral and cellular circRNAs are still unclear during FAdV-4 infection. Therefore, in our study, we have performed the comprehensive study on the cellular and viral circRNAs induced by FAdV-4 infected in LMH cells Parts of cellular and viral circRNAs were identified and confirmed by RT-PCR. The function of 10 viral circRNAs was also investigated. Our study provides novel insights about the circRNAs of host and virus during FAdV-4 infection, which may be novel therapeutic targets or biomarkers.

## Materials and Methods

### Cells, Viruses, and Plasmid

The FAdV-4 strain SDSX1 (GenBank accession no.KY636400.1) was isolated and stored in our laboratory. Leghorn male hepatoma (LMH) cells were cultured in Dulbecco’s Modified Eagle’s Medium (DMEM)/F12 (Gibco, NY, United States) supplemented with 10% FBS (Aoqing, Beijing, China) in 5% CO_2_ incubator at 37°C. The pCD2.1-ciR vector was kindly provided by Liwei Li, Shanghai Veterinary Research Institute. Fiber 2 polyclonal antibody was prepared by immunizing rabbits with the Fiber 2.

### One-Step Growth Curves and Virus Titter

LMH cells cultured in six-well plate (1.0 × 10^6^ cells/well) were infected with FAdV-4 (multiplicity of infection, MOI, of 0.1). After the virus was adsorbed at 37°C for 1 h, the supernatant was discarded, and then the cells were washed for three times with phosphate-buffered saline (PBS). The supernatant of the cells was harvested at 6-, 12-, 24-, 36-, 48- and 60 h post-infection (hpi) respectively. The median tissue culture infective dose (TCID_50_) was determined using a microtitration assay according to the method of Reed and Muench (1938).

### Indirect Immunofluorescence Assay

LMH cells infected or uninfected with FAdV-4 were fixed with 4% paraformaldehyde for 25 min and subsequently permeabilized with 0.1% Triton X-100 (Sigma, St. Louis, MO, United States) for 10 min. After blocking with 5% bovine serum albumin for 2 h, the cells were incubated with rabbit anti-Fiber 2 polyclonal antibody, followed by goat anti-Rabbit FITC (Solarbio, Beijing, China). The fluorescence signal was examined using a fluorescence microscope.

### Western Blotting

FAdV-4 infected and uninfected cell lysates were obtained using RIPA lysis buffer (Beyotim, Shanghai, China) containing 1 mM phenylmethylsulfonyl fluoride and 1 mg/ml of protease inhibitor cocktail (Roche). After centrifuging at 12,000 ×*g* for 10 min, the supernatants of cell lysates were mixed with 5× sodium dodecyl sulfate-polyacrylamide gel electrophoresis (SDS-PAGE) loading buffer (Beyotime, Shanghai, China) and then boiled for 5 min. The proteins were separated by SDS-PAGE and transfected onto a nitrocellulose membrane. The membranes were blocked with 5% skim milk for 2 h and then incubated with the indicated antibodies for 1 h at room temperature. After washing three times, the membranes were incubated with horseradish peroxidase (HRP)-conjugated Goat anti-Rabbit IgG (H + L; 1:5,000) for 1 h at room temperature. The membranes were visualized by treating with Pierce ECL WB substrate (Thermo Fisher Scientific). For the quantification of target proteins, their levels were normalized to the levels of β-actin.

### Infection and RNA Extraction

LMH cells cultured in 10 cm-dishes were infected with FAdV-4 at MOI 0.1. After 36 hpi, the total RNAs of the cells were extracted using Trizol (Takara, Beijing, China) according to the manufacturer’s instructions. The concentration and purity of the total RNAs were determined by Thermo Scientific Nanodrop NC2000 (Thermo Scientific, NY, United States), and the RNA integrity was accessed by RNA 6000 Nano Kit 5067-1511 of the Agilent 2100 Bioanalyzer (Agilent Technologies, CA, United States).

### RNA Sequencing and Data Analysis

The pooled RNA samples of the LMH cells infected or uninfected with FAdV-4 were paired-end sequenced with an IlluminaHiSeq® 2500 (Illumina Inc., San Diego, CA, United States) by Shanghai Personal Biotechnology Company Ltd. Clean reads were obtained by removing low-quality reads with quality scores <Q20 (namely, the proportion of read bases whose error rate is less than 1%) and removing the adapter reads in the raw reads. The clean reads were mapped to Gallus_gallus GRCg6a using HISAT2. The software Find_circ was then used to identify the circular RNAs. Briefly, 20-mers from both ends of these reads were extracted and aligned independently to the reference genome to find unique anchor positions within the splice sites. Anchor reads that mapped to the reversed (head-to-tail) orientation, represented circRNA splicing. Anchor alignment can be extended such that the original read sequence is completely aligned and the inferred breakpoints were flanked by GU/AG splice signals. Sequences with multiple mappings and ambiguous breakpoints were discarded. The type, chromosome distribution and length distribution of the circular RNAs identified, were statistically analyzed according to the results from the identification of Find_circ and annotation of the reference Gallus_gallus GRCg6a.

The expression level of each annotated circular RNA was normalized using a reads per kilobase of transcript per million mapped reads (RPKM; a normalized unit of transcript expression) approach, and the differentially expressed (DE) circular RNAs were identified using the DEGseq R package with the parameters of a |fold-change| > 2.0 and *p* < 0.05 being set as the threshold for selecting the DE circular RNAs.

### Validation of the RNA-seq Results Using Semiquantitative RT-PCR Analyses

Total RNAs were extracted from the infected and uninfected LMH cells using Trizol reagent. The concentration and purity of the total RNA were determined using Thermo Scientific Nanodrop NC2000. After treatment with RNase R (Epicentre, wisconsin, United States) to remove linear RNAs, cDNAs were synthesized using the First Strand cDNA Synthesis kit (Thermo Scientific, NY, United States) from 5 μg of total RNA. Amplification was conducted *via* PCR using specific divergent primers to validate the candidate circRNAs. The primers used in the study were listed in the Supplementary Table S1. Amplification program was 95°C for 5 min and 94°C for 50s, followed by 30 cycles of annealing (temperature depending on the primer set used) for 30s and extension at 72°C for 60s, with a final extension at 72°C for 10 min. PCR products were identified with 1.5% agarose gels and cloned into the pMD-18T for Sanger sequencing. The GAPDH gene was used as an internal reference.

### The Expression Patterns of Viral circRNAs

LMH cells were seeded into six-well plates at a density of 2 × 10^5^ cells per well and incubated overnight. The cells were then infected with FAdV-4 (MOI = 0.1). LMH cells infected or uninfected with FAdV-4 were collected at 6-, 12-, 24- and 36 hpi. The total RNAs of LMH were extracted by Trizol and the expression levels and expression patterns of FAdVcircRNAs (FAdVcirc_029216, FAdVcirc_011337, FAdVcirc_007095, FAdVcirc_013009, FAdVcirc_027083, FAdVcirc_012746, FAdVcirc_008682, FAdVcirc_016019, FAdVcirc_020380, and FAdVcirc_023475) were validated RT-qPCR using indicated primers (Supplementary Table S2). The GAPDH gene was used as an internal reference.

### GO and KEGG Pathway Analysis

GO analysis was performed using GOseq to annotate the genes under the category of cellular component, biological process and molecular function.^1^ Kyoto encyclopedia of genes and genomes pathway enrichment using hypergeometric test was also conducted to predict the involvement of cellular pathways targeted by circRNAs expressed by host during FAdV-4 infection. The pathways of GO and KEGG with the corrected *p* < 0.05 were chosen to be significantly enriched.

### Prediction of Target miRNAs for Candidate circRNAs

There was a study shown that circRNAs could act as miRNA sponges to regulate gene expression. Therefore, the miRanda software was used to predict the miRNAs targeted by circRNAs.^2^ And, the Cytoscape v3.4 software was used to construct the circRNA-miRNA interaction network.

### Establishment of an *in vitro* circRNA Synthesis System

To investigate the function of the viral circRNAs identified from LMH cells infected with FAdV-4, the circRNA sequences (the primers used for amplification were listed in the Supplementary Table S3) were cloned into pCD2.1-ciR by homologous recombination with the NEBuilder® HiFi DNA Assembly Master Mix (New England Biolabs; Ipswich, MA) according to the manufacturer’s instructions. The LMH cells cultured in six-well plates were transfected with 2 μg of pCD2.1-circRNA, respectively. 24 h post-transfection (hpt), the LMH cells were infected FAdV-4 with 0.1 MOI and the supernatant was harvested at 36 hpi, and the cells were lysed using RIPA lysis buffer. Viral titers in the supernatants were determined using a microtitration assay. The amount of the Fiber 2 protein was detected in cell lysates by western blotting using a rabbit anti-Fiber 2 polyclonal antibody.

### Statistical Analysis

All experiments mentioned above were performed with three independent experiments. Statistical significance was analyzed using *t-*tests. The data shown are the means ± standard variations (SD) of three independent experiments. Values of *p* less than 0.05 were considered as statistically significant.

## Results

### Identification of circRNAs

**Figure 1:**
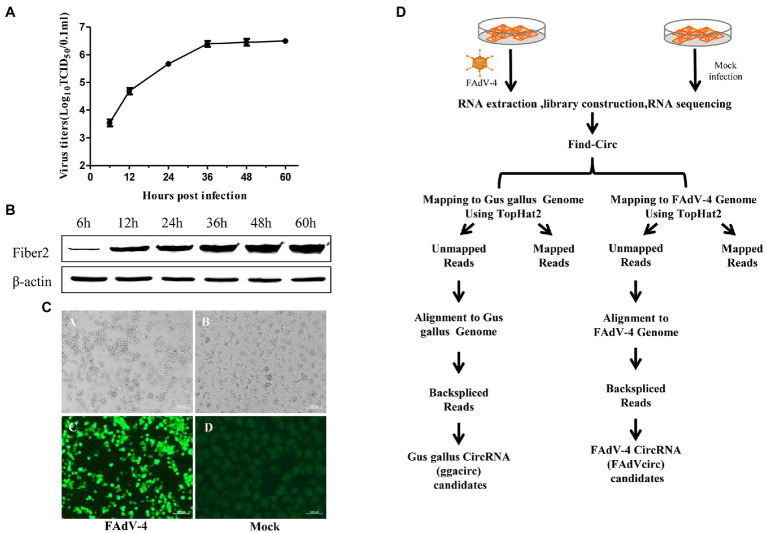
Experimental schema for the identification of cellular and viral circRNA candidates in mock and FAdV-4 infected LMH cells and characterization of mock and FAdV-4 infected LMH cells. **(A)** LMH cells were infected with FAdV-4 at a MOI of 0.1; viral supernatants collected from infected LMH cells at the indicated time points were then titrated by TCID_50_. **(B)** Analysis the expression of Fiber2 protein in LMH cells infected with FAdV-4 at different times. **(C)** FAdV-4 induced cytopathic effect in LMH cells at 36 h and the IFA result. **(D)** Flowchart demonstrates how the high-throughput sequencing data were analyzed and how cellular and viral circRNAs were identified.

The virus titers and the expression level of Fiber 2 almost reached the highest value at 36 hpi (Figures 1A,B). The indirect immunofluorescence assay (IFA) result of the LMH cells infected FAdV-4 at 36 hpi indicated that almost all the cells were infected (Figure 1C). So, the LMH cells infected FAdV-4 at 36 hpi was chosen to identify and profile of cellular circRNAs and viral circRNAs. To examine the landscape of circRNA abundances during FAdV-4 infection, RNAs from mock and FAdV-4 infected cells were isolated from several distinct pools (Figure 1D).

### Characterization of Circular RNAs

**Figure 2:**
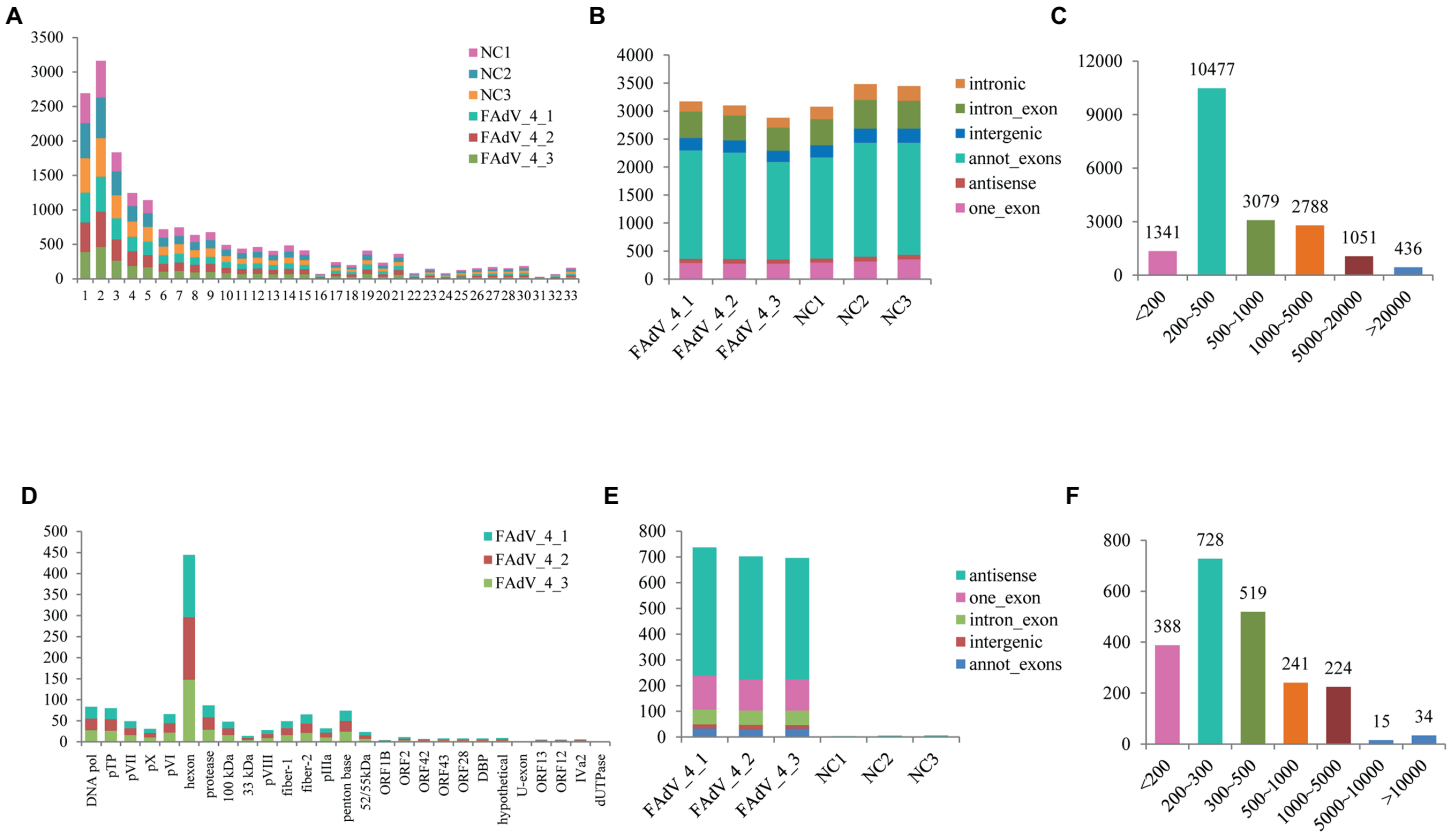
Characterization of identified circRNAs. **(A)** Quantitative distribution of ggacircrna (circRNA encoded by chicken genome) on different chromosome. **(B)** The type of identified ggacircRNAs in the six samples. **(C)** The distribution of identified ggacircRNAs sequence length. **(D)** Quantitative distribution of FadVcircrna (circRNA encoded by FAdV-4 genome) on different coding sequences (CDSs). **(E)** The type of identified FAdVcircRNAs in the six samples. **(F)** The distribution of identified FAdVcircRNAs sequence length.

A total of 458,690,632 clean reads were obtained after removing adapters and poly-N, as well as other low-quality reads (Table 1). More than 80% of these reads in the mock group were mapped to the reference chicken genome.^3^ While only about 45% of the reads in the FAdV-4 infected group were mapped to the reference chicken genome, and about 0.03% of the reads were mapped to the FAdV-4 reference genome (KY636400.1). And 21,258 candidate circRNAs were totally identified from 6 samples, 19,154 of them are encoded by the chicken genome, and the remaining 2,104 are encoded by the virus genome. Most of ggacircRNAs (encoded by the chicken genome circRNAs) were distributed on chromosome 2, followed by chromosomes 1 and 3, and least distributed on chromosome 31 (Figure 2A). The 6 cellular circular RNAs types identified based on the mapping of the circular RNAs to the chicken genome indicated that annot_exons were the most common sequences identified, accounting for 61 and 58% of the circRNAs in the NC group and FAdV-4 group, respectively. It was followed by intron_exon circRNAs. The remaining circRNAs types belong to one_exon, intergenic, antisense and intronic sequences (Figure 2B). The number of ggacircRNA with a length of 200–500 bp is the largest, with 10,477 pieces, followed by 500–1,000 bp, with 3,079 pieces (Figure 2C). The position of FAdVcircRNAs (encoded by the virus genome) on FAdV-4 genome were mainly distributed on hexon protein (Figure 2D). There were only five types of viral circRNAs and the antisense circRNAs were the most common sequences identified, followed by one_exon circRNAs. The amount of other types of circRNAs is relatively small, including annot_exons, intron_exon, and intergenic sequences (Figure 2E). The length of FAdVcircRNA was mostly between 1 and 5,000 bp, and the length of 200–300 bp was the largest, with 728 pieces, followed by 300–500 bp, with 519 pieces (Figure 2F).

**Table 1:** Summary of RNA-seq data sets from mock- and FadV-4-infected LMH cells.

Sample	Total no. of reads	Mapping ratio to chicken genome (%)	No. of cellular circRNAs (ggacircRNA)	Mapping ratio to FadV-4 genome (%)	No. of viruses circRNAs
NC1	78,482,372	69,682,183 (88.79%)	3,078	27(0%)	3
NC2	80,915,188	72,423,264 (89.51%)	3,482	19(0%)	4
NC3	77,503,978	68,641,446 (88.57%)	3,444	11(0%)	6
FAdV_4_1	74,177,560	34,252,986 (46.18%)	3,171	23,373(0.03%)	722
FAdV_4_2	74,905,602	33,545,569 (44.78%)	3,100	24,555(0.03%)	686
FAdV_4_3	72,705,932	3,258,056 (45.74%)	2,879	21,114(0.03%)	683
Total			19,154		2,104

### Analysis of Differentially Expressed Cellular circRNAs

**Figure 3:**
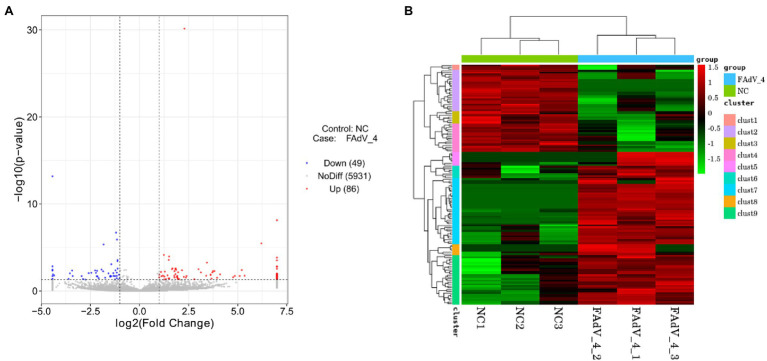
Analysis of differentially expressed circRNAs. **(A)** The volcano map in Negative Control groups compared to FAdV-4 groups. The red dots correspond to upregulated circRNAs, the blue dots correspond to downregulated circRNAs and the gray dots represent the circRNAs without statistically significant difference. The vertical gray lines represent 2.0-fold up and down, and the horizontal gray line correspond to a *p* < 0.05 **(B)** Heat map for differentially expressed circRNAs from the control groups compared to FAdV-4 groups based on RPM of RNA-seq data (log2 scale). Clustering map shows the differential expression profile of circRNAs among different groups.

The differential expression of cellular circRNA between FAdV-4 infection group and control group was identified by DEseq2, based on |log2 (fold change) | > 1 and *p* < 0.05. There were 135 DE cellular circRNAs (86 upregulated circRNAs and 49 downregulated circRNAs) obtained in the FAdV-4 groups compared to the control groups (Figure 3A). The heat map shows significantly different expressions of circRNAs between the two groups. There are nine groups in the heat map. The circRNAs in groups 2, 4, 7, and 9 have a relatively concentrated expression trend (Figure 3B).

### GO and KEGG Enrichment Analysis of Host Genes

**Figure 4:**
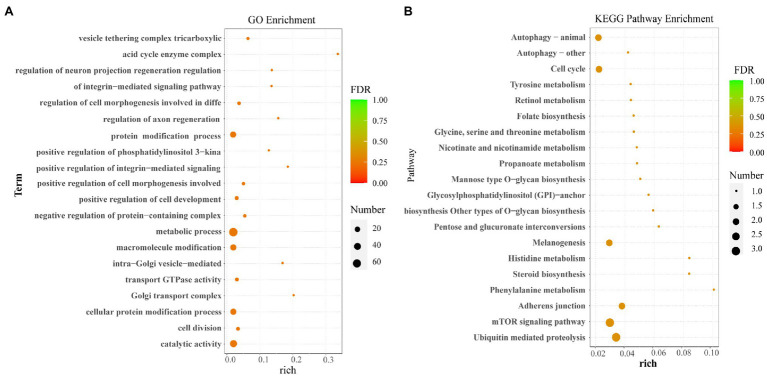
Functional enrichment analysis. **(A)** The GO enrichment analysis for host genes of the differentially expressed circRNAs. **(B)** The KEGG pathway analysis for host genes of the differentially expressed circRNAs.

The GO enrichment and KEGG pathways analysis of the host genes for DE circRNAs were performed to further study the potential function of circRNAs. The GO enrichment analysis showed that most host genes were significantly enriched in protein modification process, the cell metabolic process, macromolecule modification, and catalytic activity process (Figure 4A). Meanwhile, KEGG pathways analysis indicated that most host genes of DE circRNAs were significantly assigned to autophagy (animal), cell cycle, melanogenesis, adherens junction related pathways, and mTOR signaling pathway and ubiquitin mediated proteolysis (Figure 4B).

### Verification of Cellular DE circRNAs

**Figure 5:**
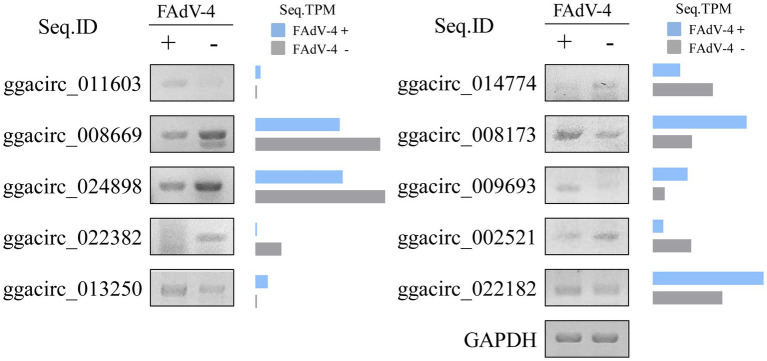
Validation of ggacircRNAs. (A) ggacircRNA was detected by Semi-Quantitative RT-PCR using divergent primers. The right histogram represents the expression level of RNA-seq results. Ten ggacircRNAs (ggacirc_011603, ggacirc_008669, ggacirc_024898, ggacirc_022382, ggacirc_013250, ggacirc_014774, ggacirc_008173, ggacirc_009693, ggacirc_002521, and ggacirc_022182) were selected for validation. The GAPDH gene was used as an internal reference.

**Figure 6:**
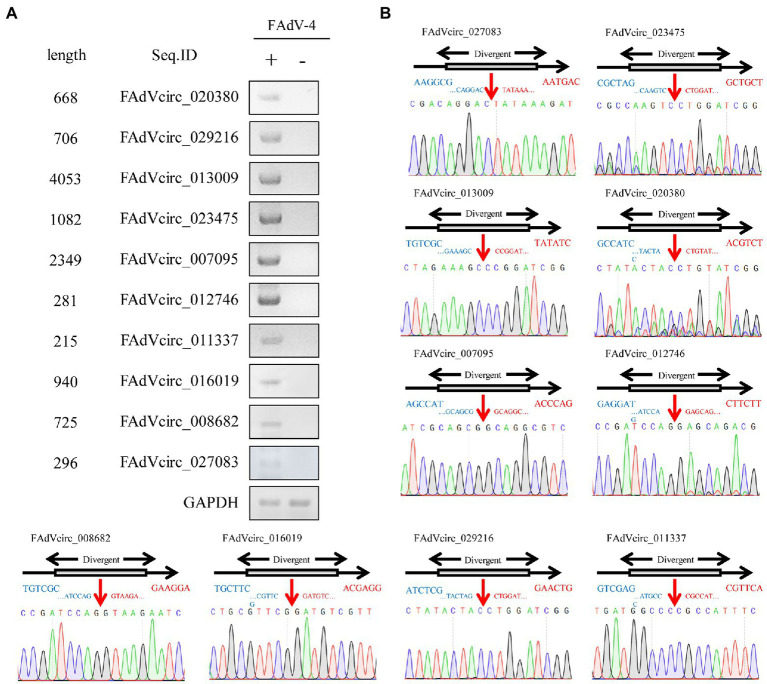
Validation of FAdVcircRNAs. **(A)** FAdVcircRNAS were detected by semiquantitative RT-PCR using divergent primers. Ten FAdVcircRNAs (FAdVcirc_020380, FAdVcirc_027083, FAdVcirc_029216, FAdVcirc_013009, FAdVcirc_023475, FAdVcirc_007095, FAdVcirc_012746, FAdVcirc_011337, FAdVcirc_016019, and FAdVcirc_008682) were selected for validation. **(B)** The expected PCR product was Sanger sequenced and the junction site was confirmed. The red arrow on the DNA sequencing peak diagram represents the junction site for the viral circRNAs.

The above circRNAs analysis was based on transcriptome sequencing (RNA-Seq) and bioinformatics. However, many factors, such as template switching, sequence homology, and degenerate sequences at exon boundaries, may lead to false positive in such a prediction. To confirm the reliability of RNA-seq results, outward-facing primers were used to amplify the back-splice junction of 10 cellular and 10 viral circRNAs by Semiquantitative PCR (Table 2). The results showed that the amount of ggacirc_011603, ggacirc_013250, ggacirc_008173, ggacirc_009693, and ggacirc_022182 were increased and ggacirc_008669, ggacirc_024898, ggacirc_022382, ggacirc_014774, and ggacirc_002521 were decreased after infected with FAdV-4 which consistent to the RNA-seq results. The bar chart on the right represents the upregulated and downregulated trends in the RNA-seq results (Figure 5). Viral circRNAs encoded by FAdV-4 (FAdVcirc_029216, FAdVcirc_011337, FAdVcirc_007095, FAdVcirc_013009, FAdVcirc_027083, FAdVcirc_012746, FAdVcirc_008682, FAdVcirc_016019, FAdVcirc_020380, and FAdVcirc_023475) were validated in FAdV-4 infected group which consistent to the RNA-seq results (Figure 6A). The sequencing results showed that the locations of the cleavage site of the 10 viral circRNA were consistent with the high-throughput sequencing (Figure 6B). Collectively, these results further confirm that these viral circRNAs were encoded by the FAdV-4 genome. And, these data further confirmed the authenticity and accuracy of sequencing data.

**Table 2:** List of circRNAs validated by divergent primers.

Name	Positions	Strand	Spliced_length	Source Gene Description
FAdVcirc_012746	AUO29782.1	+	281	Hexon protein
FAdVcirc_011337	AUO29778.1	−	215	Penton base
FAdVcirc_008682	AUO29775.1	+	725	pTP protein
FAdVcirc_013009	AUO29774.1	+	4,053	DNA pol protein
FAdVcirc_029216	AUO29775.1	+	706	pTP protein
FAdVcirc_007095	AUO29775.1	+	2,349	pTP protein
FAdVcirc_016019	AUO29774.1	+	940	DNA pol protein
FAdVcirc_010380	AUO29775.1	+	668	pTP protein
FAdVcirc_023475	AUO29775.1	+	1,082	pTP protein
FAdVcirc_027083	AUO29771.1	+	296	ORF13
ggacirc_011603	ENSGALG00000009300		301	NUMB
ggacirc_008669	ENSGALG00000013858		307	CEP76
ggacirc_024898	ENSGALG00000012200		283	GCH1
ggacirc_022382	ENSGALG00000049652		5,355	C-factor-like
ggacirc_013250	ENSGALG00000051188		11,759	F10 alpha
ggacirc_014774	ENSGALG00000036556		423	MORC4
ggacirc_008173	ENSGALG00000052101		606	-;-
ggacirc_009693	ENSGALG00000010106		456	ERI3
ggacirc_002521	NA		1,175	-;-
ggacirc_022182	ENSGALG00000047347		299	-;-

### Alternative Splicing Events Within FAdVcircRNAs

**Figure 7:**
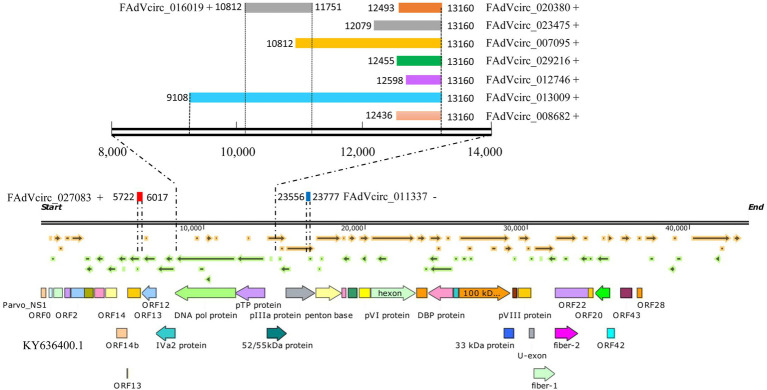
The positions of FAdVcircRNAs in the FAdV-4 genome. The symbol “+” “−” represents the distribution of FAdVcircRNA on the sense or antisense strands. The colored rectangle represents the position of circRNA in the genome, and the numbers on the left and right sides represent the position of the starting point and the ending point in the genome, respectively. The figure below represents the genomic structure of FAdV-4.

According to the results of sequence, the splicing events of viral circRNA were analyzed. The figure shows the locations of the verified viral circRNAs in the genome (some details involved can be found in Table 2). Seven FAdVcircRNAs (FAdVcirc_013009, FAdVcirc_007095, FAdVcirc_023475, FAdVcirc_008682, FAdVcirc_029216, FAdVcirc_020380, and FAdVcirc_012746) were derived from the sense strand of the FAdV-4 genome with the same end at position 13,160 and start from positions 9,108, 10,812, 12,079, 12,436, 12,455, 12,493, and 12,598, respectively (Figure 7). All seven circRNAs were located at pTP protein and DNA pol protein. Two FAdVcircRNAs (FAdVcirc_027083 and FAdVcirc_016019) started at 5722, 10812 and end at 6017, 11751 located at DNA pol protein and ORF13, respectively. The only one antisense FAdVcircRNA (FAdVcirc_011337) started at 23556 and end at 23777, which located at penton protein.

### Expression Patterns of Viral circRNAs

**Figure 8:**
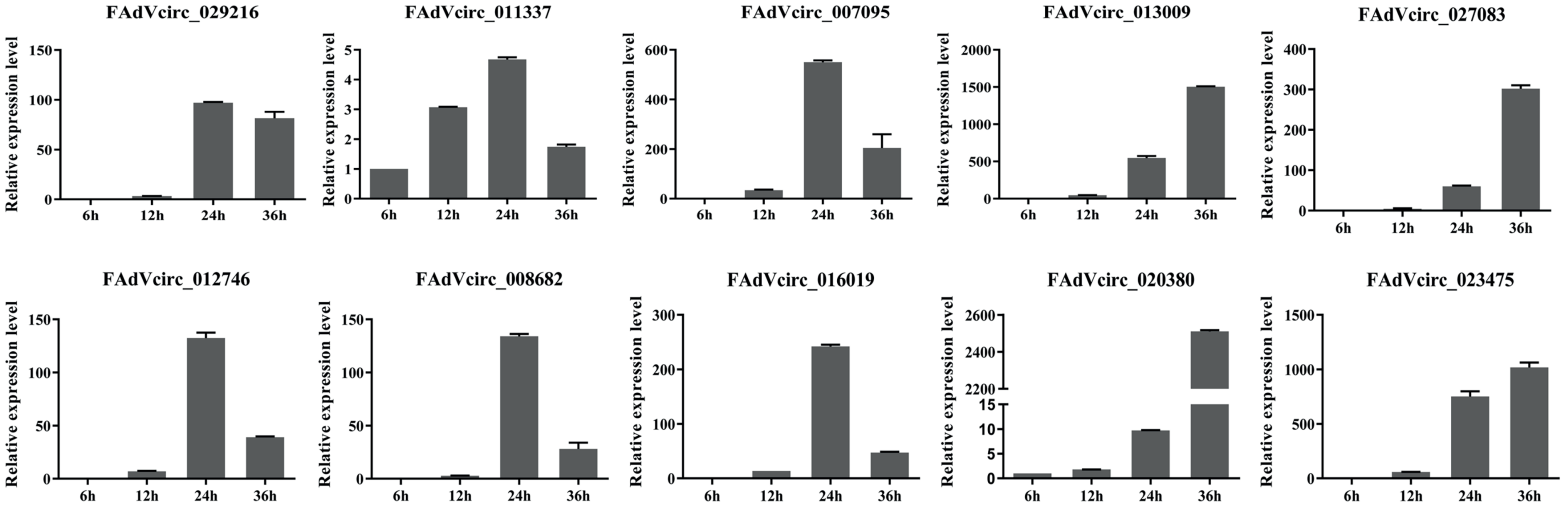
The expression levels of FAdVcircRNAs in LMH cells infected with FAdV-4 at different times. Ten FAdVcircRNAs (FAdVcirc_029216, FAdVcirc_011337, FAdVcirc_007095, FAdVcirc_013009, FAdVcirc_027083, FAdVcirc_012746, FAdVcirc_008682, FAdVcirc_016019, and FAdVcirc_020380, FAdVcirc_023475) were detected in LMH cells infected with FAdV-4 at 6, 12, 24, and 36 hpi.

In order to investigate the expression patterns of viral circRNAs during the virus life cycle, the 10 viral circRNAs were investigated by RT-qPCR at different time points after infection. The data showed that there were two expression patterns of viral circRNAs. Six circRNAs (FAdVcirc_029216, FAdVcirc_011337, FAdVcirc_007095, FAdVcirc_012746, FAdVcirc_008682, and FAdVcirc_016019) reached the peak within 24 hpi, showing a trend of first rising and then falling. While the other four circRNAs (FAdVcirc_013009, FAdVcirc_027083, FAdVcirc_020380, and FAdVcirc_023475) showing a persistent upward trend. Moreover, the expression of FAdVcirc_013009, FAdVcirc_020380, and FAdVcirc_023475 increased more than 1,000 folds at 36 h compared with 6 h (Figure 8).

### CircRNA-miRNA Target Prediction

**Figure 9:**
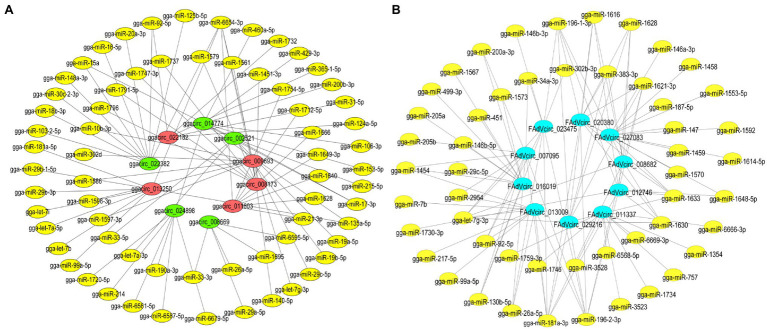
The interaction network of circRNA-miRNA. **(A)** The interaction network of the 10 DE ggacircRNAs and their target miRNAs. The red oval represents upregulated circRNA, the green oval represents downregulated circRNA, and the yellow oval represents miRNA. **(B)** The circRNA-miRNA interaction network of 10 predicted and verified FAdVcircRNA. The bule oval represents circRNA, and the yellow oval represents miRNA.

Since circRNAs regulate target genes of miRNAs by acting as miRNA sponges, the competitive interactions of 10 viral and 10 cellular circRNAs between the miRNAs were analyzed. The circRNA-miRNA network revealed that 10 viral circRNAs interacted with 814 differentially miRNAs and 51 miRNAs were shown in the network, which are highly reliable predicted and have been reported in other articles (Figure 9A). While in DE ggacircRNAs, the upregulated and downregulated top five circRNAs verified by semiquantitative RT-PCR were taken for target prediction, respectively (Figure 9B). The circRNA-miRNA target prediction results indicated that a single circRNA can target multiple miRNAs. On the contrary a single miRNA can be targeted by different circRNAs.

### Effect of FAdVcircRNAs on Virus Replication

**Figure 10:**
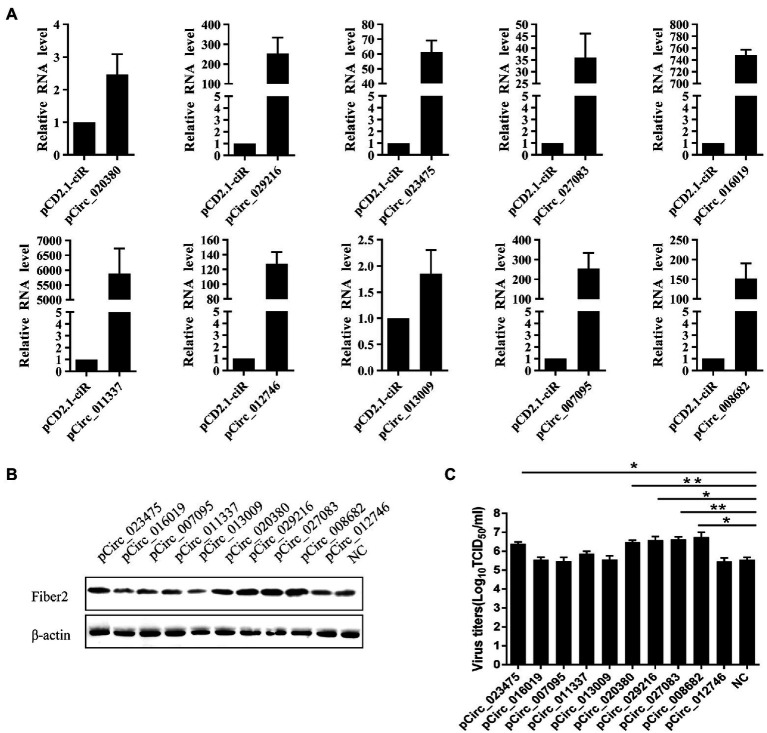
LMH cells were transfected with empty pCD2.1-ciR plasmid or overexpression plasmid, and infected with FAdV-4 at 24 hpt. **(A)** The mRNA level of circRNAs was detected at 36 hpi by RT-qPCR. GAPDH was used as the reference gene. **(B)** The expression level of FAdV-4 Fiber 2 protein was examined at 36 hpi by western blot. β-actin was used as the reference gene. **(C)** Virus titers in supernatants were measured at 36 hpi. All data are presented as the means ± SD of three independent experiments. *p < 0.05 and **p < 0.01.

In order to investigate the effect of FAdVcircRNAs on virus replication, 2 μg of pCD2.1-FAdVcircRNAs were transfected into LMH cells and then infected with FAdV-4 at 30 hpt. The overexpression of circRNAs was verified by RT-qPCR, the results showed that all the FAdVcircRNAs were overexpressed successfully (Figure 10A). The replication levels of FAdV-4 were detected by virus titer and Western Blotting. Western Blotting result showed that the expression levels of Fiber 2 protein of FAdV-4 were significantly promoted in cells transfected with pCirc_023475, pCirc_020380, pCirc_029216, pCirc_027083, and pCirc_008682 (Figure 10B). The virus titer showed the similar trends to the WB (Figure 10C). Collectively, these results suggest that the pCirc_023475, pCirc_020380, pCirc_029216, pCirc_027083, and pCirc_008682 can significantly improve the replication level of virus.

## Discussion

HHS caused by FAdV-4 is one of the most common infectious diseases of chickens. The outbreak and epidemic of FAdV-4 have caused huge economic losses to the poultry industry. Hence, it is vitally important to investigate the molecular mechanism network of interactions between the FAdV-4 and host factors. Recent studies have demonstrated that endogenous circRNAs in eukaryotes regulate the activation of innate immune proteins and cells through diverse modes of action (Li and Chen, 2021). Some DNA viruses also encode circRNAs, and foreign circRNAs have been found to stimulate an innate immune response (Li and Chen, 2021). However, there have been no reports about the expression profiles of circRNAs of cellular and viruses during FAdV-4 infection.

CircRNAs, a new class of single-stranded RNAs that lack ends, are now appreciated to have important biological roles. The formation of circRNA occurs *via* ‘back-splicing’ in which the cellular spliceosomal machinery joins a downstream splice donor to an upstream splice acceptor (Ashwal-Fluss et al., 2014). Numerous studies indicated that cellular and viral circRNAs play important roles in gene expression, innate immune system, and protein activity. CircRNAs can “sponge” miRNAs by competitive binding to block miRNA interactions with mRNAs (Hansen et al., 2013) and regulate protein activation by docking in the active sites of RNA-binding proteins (Liu et al., 2019). CircRNAs have additionally been shown to modulate mRNA stability (Ng et al., 2016) and several circRNAs are themselves translated to produce novel proteins (Legnini et al., 2017; Zhao et al., 2019). While, the circRNAs encoded by viruses also regulated the replication of viruses and contribute the immune escape of the viruses (Li and Chen, 2021).

In our study, we systematically analyzed circRNA expression profiles in LMH cells infected with FAdV-4. The results showed that two sources of circRNAs were identified including cellular circRNAs and viral circRNAs. First, from the perspective of the cellular circRNAs, 21,258 candidate circRNAs were totally identified from FAdV-4 and mock-infected LMH cells. And most of the cellular circRNAs located at chromosome 2 and the main type of circRNA were annot_exons. The lengths of the cellular circRNAs are concentrated in 200–5,000 bp. From the difference analysis of cellular circRNA, we found that 135 circRNAs were DE during FAdV-4 infections, as compared to non-infected LMH cells. Among these, 86 circRNAs were upregulated and 49 were downregulated. According to the different expression patterns of circRNAs, it can be divided into nine groups. The DE circRNAs may play an important role in host antagonism of viral infection. Studies had demonstrated that circRNAs exert antiviral effects through different biological processes in different hosts. During infection of influenza virus, a circRNA, AIVR, was upregulated to exerting its antiviral effect by absorbing miRNA and promoting the expression of CREBBP to facilitate IFN-β production (Qu et al., 2021). While, circTNFAIP3, which is distributed and expressed widely in various tissues after deltacoronavirus infection promoted deltacoronavirus replication by reducing the apoptosis (Du et al., 2021). The GO and KEGG analysis revealed that the cicrRNAs related to multiple progresses of cells, such as the process of protein modification, metabolic process, autophagy, and ubiquitin-mediated proteolysis. Therefore, we believe that these circRNAs we screened may antagonize or promote virus infection through the above biological processes. Ten circRNAs were further verified by semiquantitative RT-PCR, and their change trends were completely consistent with RNA sequencing. The DE cellular circRNAs must be associated to multiple biological processes. And, for the host, it may regulate virus infection and replication by the DE circRNAs. However, the molecular mechanism of the DE circRNAs on virus infection needs further investigation. Through the prediction analysis of the correlation between circRNA and miRNA, it is found that these 10 circRNAs can target multiple miRNAs, such as gga-let-7b, gga-let-7i, gga-miRNA-26a, gga-miRNA-181a-5p, and gga-miRNA-21 downstream predicted by cellular circRNAs (Figure 9A). The gga-let-7b and gga-let-7i were involved in multiple pathways including signaling pathways, such as MAPK, TGF-beta, Notch, Wnt, mTOR, Cell cycle, P53, and Jak–STAT (Ji et al., 2017). MiRNA-26a can target NEK6 and suppresses Marek’s disease lymphoma cell proliferation (Li et al., 2014). gga-miR-181a-5p can enhance FAdV-4 replication and negatively regulate STING mRNA and protein expression levels to inhibit type I IFN and inflammatory cytokine production (Yin et al., 2021). So the cellular circRNA may antagonize viral replication by targeting gga-miRNA-181a-5p. While, the gga-miRNA-21 could suppress IBDV replication through downregulating IBDV VP1 expression at translational level (Wang et al., 2013). All of these studies suggested that cellular circRNAs regulate multiple processes of cells by targeting miRNAs then achieving the role of defense against pathogenic infection.

Second, from the perspective of the viral circRNAs, we identified 2,104 viral circRNAs in FAdV-4 infected LMH cells, which consist to the previous study that DNA virus can express circRNA (Zhao et al., 2019; Choudhary et al., 2021; Tan and Lim, 2021). Our results revealed that most of the viral circRNA expressed by FAdV-4 were located at hexon protein, and the length is concentrated in 200–1,000 bp. The actual existence of 10 representative viral circRNAs was validated by semiquantitative RT-PCR using divergent primers and Sanger sequencing. Interestingly, among the 10 viral circRNAs, seven of circRNAs located at pTP and DNA pol protein possessed the same 3′ terminal cleavage site (Figure 7). CircRNAs appear as intermediates or final product of RNA processing pathways which were generated *via* an alternative splicing mechanism within the host cells. The sequence of the cleavage sites in the genome of the virus may relate to the cleavage specificity and cleavage frequency which induces the same cleavage sites. The circularization process protects the viral genome from digestion by intracellular exonucleases, reduces the immunogenicity of infected cells, and helps immune escape, and thus promoting the replication of FAdV-4 (Petkovic and Müller, 2015). In addition to, the data showed that these viral circRNAs had low expression levels between 0 and 12 h and high expression levels between 24 and 36 hpi (Figure 8). This expression pattern of circRNA may be related to the replication efficiency of the virus for the virus is almost at the peak of the replication rate between 24 and 36 hpi. Moreover, we were particularly interested in the functions of the viral circRNAs encoded by the virus genome. Accumulating data indicated that circRNAs regulated the expression of target genes by mediating miRNAs or RNA-binding proteins (Cortés-López and Miura, 2016). In order to investigate the potential roles of viral circRNAs, we predicted the miRNA downstream of the viral circRNAs. It is known that miRNAs not only regulate host target gene expression but also mediate viral gene products (Hussain et al., 2008). As a special class of regulatory factors in cells, miRNAs can regulate viral replication through different ways of action. FAdVcirc_013009 and FAdVcirc_011337 target the gga-miR-7475-5p, which negatively correlated with CREBZF and LFNG during FAdV-4 infection, and these genes were all associated with the host cell response to viral infection (Wu et al., 2020). It is possible that viral circRNAs sequester functions from host miRNAs or viral miRNAs in order to regulate host or viral target genes. To verify the function in promoting the replication of FAdV-4, we established an *in vitro* method for viral circRNA synthesis. When viral circRNA_023475, circRNA_020380, circRNA_029216, circRNA_027083, and circRNA_008682 were overexpressed in LMH cells, the expression levels of the Fiber 2 of FAdV-4 was increased significantly, which was consistent with the virus titer (Figures 10B,C). Two reasons may explain this phenomenon, firstly, one possible function of circRNAs is sponging viral miRNAs and preventing miRNA functions, so that some viral circRNAs facilitate the replication of FAdV-4 by targeting the miRNAs which suppress the proliferation of FAdV-4. Secondly, some viral circRNAs may be inhibit viral gene expression and reduce the immunogenicity of infected cells and contribute to the immune escape of virus then promoting the replication of the FAdV-4.

Overall, we revealed a new layer of host-virus interactions with circRNAs: FAdV-4 infection induced the differential expression of cellular circRNAs, which target different miRNAs. And, FAdV-4 can generate circRNAs *via* unknown splicing machinery in the host that promote virus replication. These findings will provide novel clues to further our understanding of FAdV-4 and provide a basis for investigating host-virus interactions.

## Data Availability Statement

The data presented in the study are deposited in the NCBI Sequence Read Archive (https://www.ncbi.nlm.nih.gov/sra/PRJNA833916) repository, accession number PRJNA833916.

## Author Contributions

KZ designed the experiments. X-NL, X-RG, YH, TT, JS, B-SL, and W-CZ performed the experiments. KZ and X-NL analyzed the data and wrote the manuscript. W-ZY made constructive comments to the experiments. All authors contributed to the article and approved the submitted version.

## Funding

This work was supported by the Natural Science Foundation of Hebei Province (grant no. 2020204045), the Talents Introduction Projects of Hebei Agricultural University (grant no. YJ201945), and the Precision Animal Husbandry Discipline Group Construction Project of Hebei Agricultural University (grant no. 1090064).

## Conflict of Interest

The authors declare that the research was conducted without any commercial or financial relationships that could be construed as a potential conflict of interest.

## Publisher’s Note

All claims expressed in this article are solely those of the authors and do not necessarily represent those of their affiliated organizations, or those of the publisher, the editors and the reviewers. Any product that may be evaluated in this article, or claim that may be made by its manufacturer, is not guaranteed or endorsed by the publisher.

## Footnotes

^1^ http://www.geneontology.org/

^2^ http://www.mirbase.org/index.shtml

^3^ http://ftp.ensembl.org/pub/release-103/fasta/gallus_gallus/dna/Gallus_gallus.GRCg6a.dna.toplevel.fa.gz
