# Supplementary material for: The Cellular and Viral circRNAs Induced by Fowl Adenovirus Serotype 4 Infection
Source: Front Microbiol. 2022 Jun 2;13:925953. doi: 10.3389/fmicb.2022.925953 (PMC9201442; doi:10.3389/fmicb.2022.925953)
Supplement: Supplementary file 1 [file Data_Sheet_1.docx]

Table S1. Primer sequences for verifying circRNA

| CircRNA ID | Primers | Sequence | Product length（bp） |
| --- | --- | --- | --- |
| ggacirc_011603 | Forward | GTTTGTTTTCGACAGGATCGGTT | 301 |
|  | Reverse | CAGGACCACTTCCAGAAGGAGC |  |
| ggacirc_024898 | Forward | CTCGCGAGGATTGTGGAGATATACAG | 283 |
|  | Reverse | CTTGCTGAGGCCAAGTACTTGTTT |  |
| ggacirc_013250 | Forward | TCCTCAAGTTCTGAACGCAGACAG | 421 |
|  | Reverse | TCGGGTACGTGGATGGCGACCTCT |  |
| ggacirc_008173 | Forward | GTGACCCGGACTTCCGGCAGCCTT | 616 |
|  | Reverse | TGCCAGGACCTGTGGGGTACC |  |
| ggacirc_002521 | Forward | TCAGGCAACACAAGGAATTGCAC | 270 |
|  | Reverse | TGTACTTACTGCCCCAGTTGCAA |  |
| ggacirc_008669 | Forward | TCAGGAACCAGATCCTCTGCCTG | 307 |
|  | Reverse | AGATGTTCCAGAAAAGCTTTTCCACC |  |
| ggacirc_022382 | Forward | CACTTGACACAGAGACACTGGACAAC | 376 |
|  | Reverse | CAACACAGGGAAGCCCCTACC |  |
| ggacirc_014774 | Forward | GATAGTTCCAGAAAGTGACTACTCAC | 422 |
|  | Reverse | GGTCCAGTCTCTCTTGTTTTTTATACCC |  |
| ggacirc_009693 | Forward | TTCAGCCTGTGGTGCATCCCCA | 456 |
|  | Reverse | CATACATGTGAAAGGTGGATTCGAT |  |
| ggacirc_022182 | Forward | GCTTGTGCCTGTAATCCAAGCG | 299 |
|  | Reverse | CACCGTGCCCGGCAAGAGGTT |  |
| FAdVcirc_010380 | Forward | GCCATCCCGCTTGAGGATC | 238 |
|  | Reverse | TTCTACCGTCTGCTCCTGCAGA |  |
| FAdVcirc_023475 | Forward | CGCTAGCCAGAGTACTAACGAGAC | 324 |
|  | Reverse | ACAAGTCGGGAGACATCGACGA |  |
| FAdVcirc_007095 | Forward | AGCCATCCCGCTTGAGGATC | 272 |
|  | Reverse | GTAGACGTCATCGTGTTGGGTC |  |
| FAdVcirc_029216 | Forward | ATCTCGCCATTATGGACGCAG | 323 |
|  | Reverse | CAGTTCGCCACCGCCTTCTA |  |
| FAdVcirc_012746 | Forward | CTCGCCATTATGGACGCAGGA | 252 |
|  | Reverse | GTTCAGGCGCTACGTGGAAATC |  |
| FAdVcirc_013009 | Forward | GAGGATCGTTTTCGACCGCG | 322 |
|  | Reverse | CCCGAAGAAGAGGAAGAGGAAGAA |  |
| FAdVcirc_016019 | Forward | TGTCGCTAGCCAGAGTACTAACG | 275 |
|  | Reverse | GCTCACCTGCGGCCTTTATATAG |  |
| FAdVcirc_008682 | Forward | TGTCGCTAGCCAGAGTACTAACG | 313 |
|  | Reverse | TCTCGTCACCGACTCCTTCCT |  |
| FAdVcirc_011337 | Forward | GTCGAGGGCGTTAACTCGGT | 215 |
|  | Reverse | CTATCCGGCGATGGTGAACG |  |

Table S2. Primer sequences for RT-qPCR

| CircRNA ID | Primers | Sequence |
| --- | --- | --- |
| FAdVcirc_020380 | Forward | AGGAAGGAGTCGGTGACGAG |
|  | Reverse | CCATCGACGCCTTACAGCAA |
| FAdVcirc_023475 | Forward | CATCCCGCTTGAGGATCGTT |
|  | Reverse | TCAGCGCATTACCGACAACT |
| FAdVcirc_007095 | Forward | TGAGCCAATCGTCTTCAGCG |
|  | Reverse | GATCCTTCATGCCTCGCGTC |
| FAdVcirc_016019 | Forward | GGAACTTGAGGACGCCGATG |
|  | Reverse | TCGCGTCGGCAGACTATTG |
| FAdVcirc_008682 | Forward | TCTAGCTCGCCAAAGGCTTC |
|  | Reverse | CGCCTCCACGCTATACTACC |
| FAdVcirc_012746 | Forward | GCCAATCGTCTTCAGCGAAC |
|  | Reverse | AGAGGCACGACCTGTTCCA |
| FAdVcirc_013009 | Forward | GCCAATCGTCTTCAGCGAAC |
|  | Reverse | AGCCCATTCGATTCCTCGAC |
| FAdVcirc_029216 | Forward | AGCCAATCGTCTTCAGCGAAC |
|  | Reverse | CTCGTCACCGACTCCTTCCT |
| FAdVcirc_027083 | Forward | AAGGCGCTGGAAATAGGGTC |
|  | Reverse | GTCATTTCGAGCTGCTTCCC |
| FAdVcirc_011337 | Forward | CCCACATTGCACCACTCCTT |
|  | Reverse | GGCGATGGTGAACGGCTATC |
| GAPDH | Forward | GAACATCATCCCAGCGTCCA |
|  | Reverse | CGGCAGGTCAGGTCAACAAC |

Table S3. Primer sequences for overexpressing circRNA

| CircRNA ID | Primers | Sequence | Product length（bp） |
| --- | --- | --- | --- |
| FAdVcirc_020380 | Forward | CTGTTCAATTAACG*GTACCTGAAATATGCTATCTTACAG*GTAGTATAGCGTGGAGGCGA | 668 |
|  | Reverse | ATCCCAAATTAGT*GGATCCTCAAGAAAAAATATATTCAC*CTGGATCGGAAGTGTTCGCT |  |
| FAdVcirc_023475 | Forward | CTGTTCAATTAACG*GTACCTGAAATATGCTATCTTACAG*GACTTGGCGTAAGTTGTCGG | 1082 |
|  | Reverse | ATCCCAAATTAGT*GGATCCTCAAGAAAAAATATATTCAC*CTGGATCGGAAGTGTTCGCT |  |
| FAdVcirc_007095 | Forward | CTGTTCAATTAACG*GTACCTGAAATATGCTATCTTACAG*GATGTCGTTGAACAATAGTC | 2349 |
|  | Reverse | ATCCCAAATTAGT*GGATCCTCAAGAAAAAATATATTCAC*CTGGATCGGAAGTGTTCGCT |  |
| FAdVcirc_016019 | Forward | CTGTTCAATTAACG*GTACCTGAAATATGCTATCTTACAG*GATGTCGTTGAACAATAGTC | 940 |
|  | Reverse | ATCCCAAATTAGT*GGATCCTCAAGAAAAAATATATTCAC*CGAACGCAGTCGCCAGTACG |  |
| FAdVcirc_008682 | Forward | CTGTTCAATTAACG*GTACCTGAAATATGCTATCTTACAG*CGTGTCGATTTCCACGTAGC | 725 |
|  | Reverse | ATCCCAAATTAGT*GGATCCTCAAGAAAAAATATATTCAC*CTGGATCGGAAGTGTTCGCT |  |
| FAdVcirc_012746 | Forward | CTGTTCAATTAACG*GTACCTGAAATATGCTATCTTACAG*GAGCAGACGGTAGAAGGCGG | 503 |
|  | Reverse | ATCCCAAATTAGT*GGATCCTCAAGAAAAAATATATTCAC*CTGGATCGGAAGTGTTCGCT |  |
| FAdVcirc_013009 | Forward | CTGTTCAATTAACG*GTACCTGAAATATGCTATCTTACAG*GCTTTCTAGGTGGAGTACGG | 4053 |
|  | Reverse | ATCCCAAATTAGT*GGATCCTCAAGAAAAAATATATTCAC*CTGGATCGGAAGTGTTCGCT |  |
| FAdVcirc_029216 | Forward | CTGTTCAATTAACG*GTACCTGAAATATGCTATCTTACAG*CGCCTGAACTCGCGGTAGTT | 706 |
|  | Reverse | ATCCCAAATTAGT*GGATCCTCAAGAAAAAATATATTCAC*CTGGATCGGAAGTGTTCGCT |  |
| FAdVcirc_027083 | Forward | CTGTTCAATTAACG*GTACCTGAAATATGCTATCTTACAG*TTGATCGCACTGTCGGTGAT | 206 |
|  | Reverse | ATCCCAAATTAGT*GGATCCTCAAGAAAAAATATATTCAC*GTCCTGTCGGTCCTCAGGGA |  |
| FAdVcirc_011337 | Forward | CTGTTCAATTAACG*GTACCTGAAATATGCTATCTTACAG*GGGCCATCACCCGCTGAA | 215 |
|  | Reverse | ATCCCAAATTAGT*GGATCCTCAAGAAAAAATATATTCAC*CGCCATTTCGACAGGATCT |  |
